# Simulating the effects of short-term synaptic plasticity on postsynaptic dynamics in the globus pallidus

**DOI:** 10.3389/fnsys.2013.00040

**Published:** 2013-08-08

**Authors:** Moran Brody, Alon Korngreen

**Affiliations:** ^1^The Leslie and Susan Gonda Multidisciplinary Brain Research Center, Bar-Ilan UniversityRamat Gan, Israel; ^2^The Mina and Everard Goodman Faculty of Life Sciences, Bar-Ilan UniversityRamat Gan, Israel

**Keywords:** network, neuron, short-term plasticity, facilitation, depression, metabotropic receptors, deep brain stimulation, simulation

## Abstract

The rat globus pallidus (GP) is one of the nuclei of the basal ganglia and plays an important role in a variety of motor and cognitive processes. *In vivo* studies have shown that repetitive stimulation evokes complex modulations of GP activity. *In vitro* and computational studies have suggested that short-term synaptic plasticity (STP) could be one of the underlying mechanisms. The current study used simplified single compartment modeling to explore the possible effect of STP on the activity of GP neurons during low and high frequency stimulation (HFS). To do this we constructed a model of a GP neuron connected to a small network of neurons from the three major input sources to GP neurons: striatum (Str), subthalamic nucleus (STN) and GP collaterals. All synapses were implemented with a kinetic model of STP. The *in vitro* recordings of responses to low frequency repetitive stimulation were highly reconstructed, including rate changes and locking to the stimulus. Mainly involved were fast forms of plasticity which have been found at these synapses. The simulations were qualitatively compared to a data set previously recorded *in vitro* in our lab. Reconstructions of experimental responses to HFS required adding slower forms of plasticity to the STN and GP collateral synapses, as well as adding metabotropic receptors to the STN-GP synapses. These finding suggest the existence of as yet unreported slower short-term dynamics in the GP. The computational model made additional predictions about GP activity during low and HFS that may further our understanding of the mechanisms underlying repetative stimulation of the GP.

## Introduction

The rat globus pallidus (GP), homologous of the primate and human Globus Pallidus external segment (GPe), is one of the nuclei of the basal ganglia and plays an important role in a variety of motor and cognitive processes (Kita and Kitai, 1994; Kita, 2007; Sadek et al., 2007; Goldberg and Bergman, 2011). More than 80% of the inputs reaching the GP are inhibitory. Nevertheless, the firing rate of GP neurons increases during various motor actions (Georgopoulos et al., 1983; Mink and Thach, 1991; Gardiner and Kitai, 1992; Turner and Anderson, 1997). One explanation for this seeming contradiction may be the involvement of short- term synaptic depression of the GP inhibitory synapses. Such short-term synaptic depression has been reported for inputs from the striatum to the GP (Str-GP) (Rav-Acha et al., 2005; Sims et al., 2008), for the connections from the subthalamic nucleus to the GP (STN-GP) (Hanson and Jaeger, 2002) and GP-GP synapses (Sims et al., 2008). It has been suggested that both Str-GP and GP-GP synapses undergo depression during stimulation (Rav-Acha et al., 2005; Sims et al., 2008), while STN-GP synapses display facilitation followed by depression (Hanson and Jaeger, 2002). Simultaneous weakening of inhibitory synapses and strengthening of excitatory synapses could lead to domination of excitatory inputs. This would explain the elevation of firing rate seen during motor actions.

Short-term plasticity (STP) at GP synapses could also explain an *in vitro* data set recently recorded in our lab, in which low frequency stimulation (LFS) locked the firing of GP neurons to the stimulus but had only a mild impact on their firing rate (Bugaysen et al., 2011). In contrast, high frequency stimulation (HFS) generated biphasic modulation of the firing frequency, with inhibitory and excitatory phases. Blocking synaptic transmission showed that these effects result from synaptic activity and not from direct activation of the neurons.

Due to changes in the pattern of GP activity during Parkinson's disease (Nini et al., 1995; Raz et al., 2000; Mallet et al., 2008; Bronfeld et al., 2010; Moran et al., 2012), the GP has become a target for high frequency deep brain stimulation (DBS) to treat the symptoms of the disease (Yelnik et al., 2000; Dostrovsky et al., 2002; Bar-Gad et al., 2004; Vitek et al., 2004). Stimulation of the GP may have a broad impact on the function of the basal ganglia due to its input-output connections, but the physiological effects of DBS of the GP are still unclear. Analyzing its possible effects will further our understanding of GP influence on the basal ganglia.

To shed light on the mechanisms generating GP activity in the normal and pathological state it is important to consider the effects of STP on GP dynamics. These were qualitatively explored here using a simple single compartment model. The model consisted of a GP neuron connected to a small network of neurons from the three major input sources to the GP, striatum, subthalamic nucleus and GP-GP synapses. All synapses were implemented with a kinetic model of STP and the model attempted to reproduce the previously recorded data set (Bugaysen et al., 2011).

## Methods

### Short-term synaptic plasticity in ionotropic synapses

The mathematical framework used here to implement STP dynamics has been previously described (Varela et al., 1997). This model faithfully captured physiological results although it pays no attention to the biological mechanisms underlying STP. The postsynaptic amplitude, *A*, relies on three factors, initial amplitude, *A*_0_, facilitation variable, *F*, and depression variable, *D*.

(1) $$A=A_{0}FD$$

Both *F* and *D* were initially set to 1.

The depression variable *D* was multiplied with each stimulus by a constant *d* representing the depression following a single action potential:

(2) D→Dd

Since *d* ≤ 1, *D* decreased with each action potential. After each stimulus, *D* recovered exponentially to 1 using first order kinetics with time constant τ_*D*_:

(3) $$\tau_{D}\frac{dD}{dt}=1-D$$

The facilitation variable *F* was increased with each stimulus by a constant *f* representing the facilitation following a single action potential:

(4) F→F+f

Since *f* ≥ 0, *F*, increased with each action potential. *F* was increased but not multiplied by *f* since multiplication during HFS caused *F* to grow beyond any biological proportion. After each stimulus *F* recovered exponentially to 1 using first order kinetics with time constant τ_*F*_:

(5) $$\tau_{F}\frac{dF}{dt}=1-F$$

### Type 1 metabotropic glutamate receptors

The model used to implement type 1 metabotropic glutamate receptors was based on a previous model implementing GABA-B metabotropic receptors (Destexhe et al., 1998). The model is described by the following scheme:

(6) $$R_{0}+T⇄R$$

(7) R⇄G

where binding of a neurotransmitter *T* to an inactivated receptor *R*_0_ causes the receptor to reach the active state *R*. The receptor activation leads to G-protein activation *G* which represents the increase in membrane conductance. This scheme is given by the following equations:

(8) $$\frac{d[R]}{dt}=Kf_{R}*[T]*(1-R)-Kb_{R}*R$$

(9) $$\frac{d[G]}{dt}=Kf_{G}*R-Kb_{G}*G$$

(10) $$I_{i}=g\max*G*(v-E_{i})$$

where [*R*] represents the fraction of activated receptors and [*G*] represents the fraction of activated G-proteins. With each action potential, *T* was increased from 0 to constant value *X* for amount of time *t* and then decayed exponentially back to 0 using first order kinetics with time constant τ_*T*_:

(11) $$\tau_{T}\frac{dT}{dt}=-T$$

### Parameter sensitivity analysis

To test the sensitivity of the model to variations in the value of a single parameter the simulation was performed with 100 random values of this parameter sampled from a normal distribution. Afterwards the mean trial was calculated for each parameter, and for every sampled value the distance from the mean trial was estimated using a mean square error (MSE) function:

(12) $$\chi^{2}=E\left({\left(\hat{\theta }-\theta \right)}^{2}\right)=\frac{1}{N}\sum {\left(y_{i}^{*}-y_{i}\right)}^{2}$$

### Data analysis

All simulations were carried out using Neuron 7.1, data analysis used Igor Pro 6.02A (WaveMetrics) and MATLAB R2010a (Mathworks). Firing rate and poststimulus time histogram (PSTH) were calculated for each simulation. Mean firing rate was calculated for each category using 1 s bins. The PSTH was calculated for each frequency by averaging the action potentials of every millisecond in a 100 ms time window after each stimulus. All rate and PSTH results were presented as normalized frequency relative to the prestimulus firing rate under control conditions.

## The model

**Figure 1:**
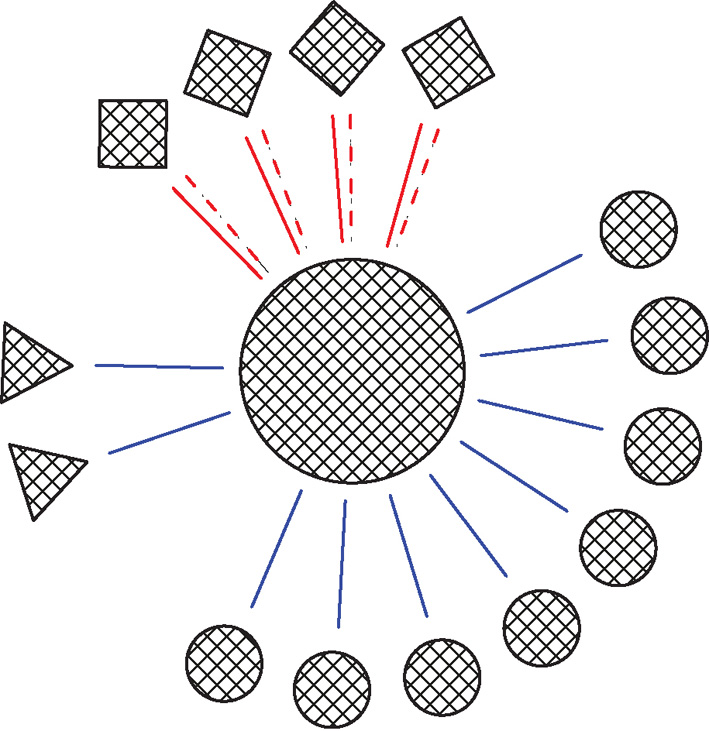
**Schema of the network.** Circles, squares and triangles represent GP, STN, and Str neurons, respectively. Blue solid lines represent ionotropic GABAergic synapses, red solid lines represent ionotropic glutamatergic synapses and red dashed lines represent metabotropic glutamatergic synapses. The ratio of the various neuron types in the figures was that in the full network.

The model consisted of one simplified postsynaptic, regular firing, neuron connected to a network of inputs including Str, STN and GP neurons (Figure 1). Different neuron types displayed different neuronal firing properties and were connected to the main postsynaptic neuron through synapses with distinctive dynamics. All the cellular and synaptic properties are described below.

### Cellular properties

All neurons consisted of a single compartment soma (diameter 96 μm). The channel dynamics in the neurons followed a modified Hodgkin–Huxley model (Hodgkin and Huxley, 1952) that was adapted to display regular firing phenotype (Pospischil et al., 2008). This model generated action potentials with amplitude of 92 mV (measured from threshold) and a half-width of 0.9 ms at 36 degrees centigrade.

The sodium conductance of this model obeyed the following equations (Pospischil et al., 2008).

(13) $$\begin{array}{l} I_{Na}={\bar{g}}_{Na}m^{3}h\left(V-E_{Na}\right)\\ \frac{dm}{dt}=\alpha_{m}(V)(1-m)-\beta_{m}(V)m\\ \frac{dh}{dt}=\alpha_{h}(V)(1-h)-\beta_{h}(V)h\\ \alpha_{m}=\frac{-0.32(V-V_{T}-13)}{\exp\left[-\left(\frac{V-V_{T}-13}{4}\right)\right]-1}\\ \beta_{m}=\frac{0.28(V-V_{T}-40)}{\exp\left[-\left(\frac{V-V_{T}-40}{5}\right)\right]-1}\\ \alpha_{h}=0.128\exp\left[-\frac{V-V_{T}-17}{18}\right]\\ \beta_{m}=\frac{4}{1+\exp\left[-\left(\frac{V-V_{T}-40}{5}\right)\right]} \end{array}$$

The potassium conductance of this model obeyed the following equations (Pospischil et al., 2008).

(14) $$\begin{array}{l} I_{K}={\bar{g}}_{K}n^{4}\left(V-E_{K}\right)\\ \frac{dn}{dt}=\alpha_{n}(V)(1-n)-\beta_{n}(V)n\\ \alpha_{n}=\frac{-0.032\left(V-V_{T}-15\right)}{\exp\left[-\left(\frac{V-V_{T}-5}{5}\right)\right]-1}\\ \beta_{n}=0.5\exp\left[-\frac{V-V_{T}-10}{40}\right] \end{array}$$

The sodium and potassium channel conductances were set to *g*_Na_ = 0.05 S/cm^2^ and *g*_K_ = 0.005 S/cm^2^ and their reversal potential to *E*_Na_ = 50 mV and *E*_K_ = −100 mV, respectively. Conductance for the leak current was set to *g*_Leak_ = 0.0001 S/cm^2^ and the reversal potential to *E*_leak_ = −65 mV. V_T_ adjusts spike threshold and was set in our model to −63 mV. Some of the models presented in Pospischil et al. (2008) contain either a slow potassium current (m-current) to introduce spike frequency adaptation or voltage-gated calcium currents to generate bursting. To keep the model of our postsynaptic neuron as simple as possible these additional channels were not inserted.

STN neurons display spontaneous firing rate at rest (Nakanishi et al., 1987; Bevan and Wilson, 1999; Do and Bean, 2003) ranging between 5 and 40 Hz (Nakanishi et al., 1987). The simulated STN neurons here were characterized by a 6 Hz firing frequency. Sixteen STN neurons were attached to the main postsynaptic neuron by a glutamatergic synapse. A stimulating electrode was inserted into ten of these neurons (Table 1).

**Table 1:** **Characteristics of the different neuron types constituting the input network**.

**Cell type**	**Spontaneous firing rate (Hz)**	**Number of cells attached to the main GP cell**	**Number of stimulated cell**
STN	6	16	10
GP	13	29	6
Str	–	8	8

GP neurons have also been found to exhibit spontaneous firing rate at rest (Cooper and Stanford, 2000; Bugaysen et al., 2010); in this model the GP neurons connected to the principle cell were adjusted to display a 13 Hz firing frequency corresponding to our previous recordings (Bugaysen et al., 2010). Twenty-nine neurons were attached to the main postsynaptic neuron. A stimulating electrode was inserted into six of these neurons (Table 1). Str neurons are quiescent at rest (Delong, 2000). Thus our model included only Str neurons into which a stimulating electrode was attached. Eight Str neurons were attached to the main postsynaptic neuron and stimulated during the simulation (Table 1).

### Synaptic properties

STN-GP synapses undergo facilitation followed by fast depression (Hanson and Jaeger, 2002). Additionally, the simulations predicted the involvement of slower synaptic dynamics including augmentation and slow depression. Thus, implementation of these synaptic properties resulted in extension of Equation (1) as follows:

(15) $$A=A_{0}*f*d_{\text{fast}}*a*d_{\text{slow}}$$

The fast depression and facilitation values were set at: *d*_fast_ = 0.9, τ_*D*_fast__ = 491 ms, *f* = 0.4 τ_*F*_ = 170 ms, based on previous findings (Hanson and Jaeger, 2002), while the augmentation and slow depression were set at: *d*_slow_ = 0.9975 τ_*D*_slow__ = 250,000 ms, *a* = 0.03, τ_*A*_ = 8000 ms (Table 2). Activation of these synapses produced an EPSP of 0.42 mV.

**Table 2:** **Synaptic parameters**.

**Cell Type**	**Parameter**	**Value**	**Decay time (ms) (τ)**
STN	*f*	0.4	170
	*d*_fast_	0.9	491
	*a*	0.03	8000
	*d*_slow_	0.9975	250,000
GP	*d*	0.998	20,000
Str	*d*	0.8	600

The STN-GP synapses were supplemented with type 1 metabotropic glutamate receptors (mGluRs1) using Equations (8–11) and the rate equations were adjusted with the rate constants *Kf*_*R*_ = 20 ms, *Kb*_*R*_ = 10,000 ms, *Kf*_*G*_ = 10 ms, *Kb*_*G*_ = 30 ms. With each action potential, *T* was set at *T* = 0.2, for 0.28 ms, afterwards decaying exponentially to 0 with time constant τ_*T*_ = 0.01 ms, and the maximal conductivity was *g*max = 0.06 pS.

Sims et al. (2008) reported that GP-GP synapses undergo a minor fast depression. This was not implemented in our model. However, our simulations predicted that this synapse should display slow depression, which was therefore implemented using Equation (1) with facilitation factors (*f*, τ_*F*_) subtracted. The depression constants were set at *d* = 0.998, τ_*D*_ = 20000 ms (Table 2).

The Str-GP synapses are inhibitory synapses that undergo rapid depression during stimulation (Rav-Acha et al., 2005). These synapses were also implemented using Equation (1) without facilitation. The depression constants for these synapses were set at *d* = 0.998, τ_*D*_ = 20000 ms, based on Rav-Acha et al. (2005) (Table 2).

## Results

STP dynamics reported for Str-GP, STN-GP and GP-GP synapses were implemented in the attempt to qualitatively simulate a previously recorded *in vitro* data set (Bugaysen et al., 2011). Str-GP synapses were characterized by fast depression with a time constant of 600 ms (Rav-Acha et al., 2005) and STN-GP synapses were characterized by facilitation with a time constant of 170 ms, followed by depression with a time constant of 491 ms (Hanson and Jaeger, 2002). Since GP-GP synapses undergo a minor and insignificant depression (Sims et al., 2008), they were not implemented with STP kinetics.

**Figure 2:**
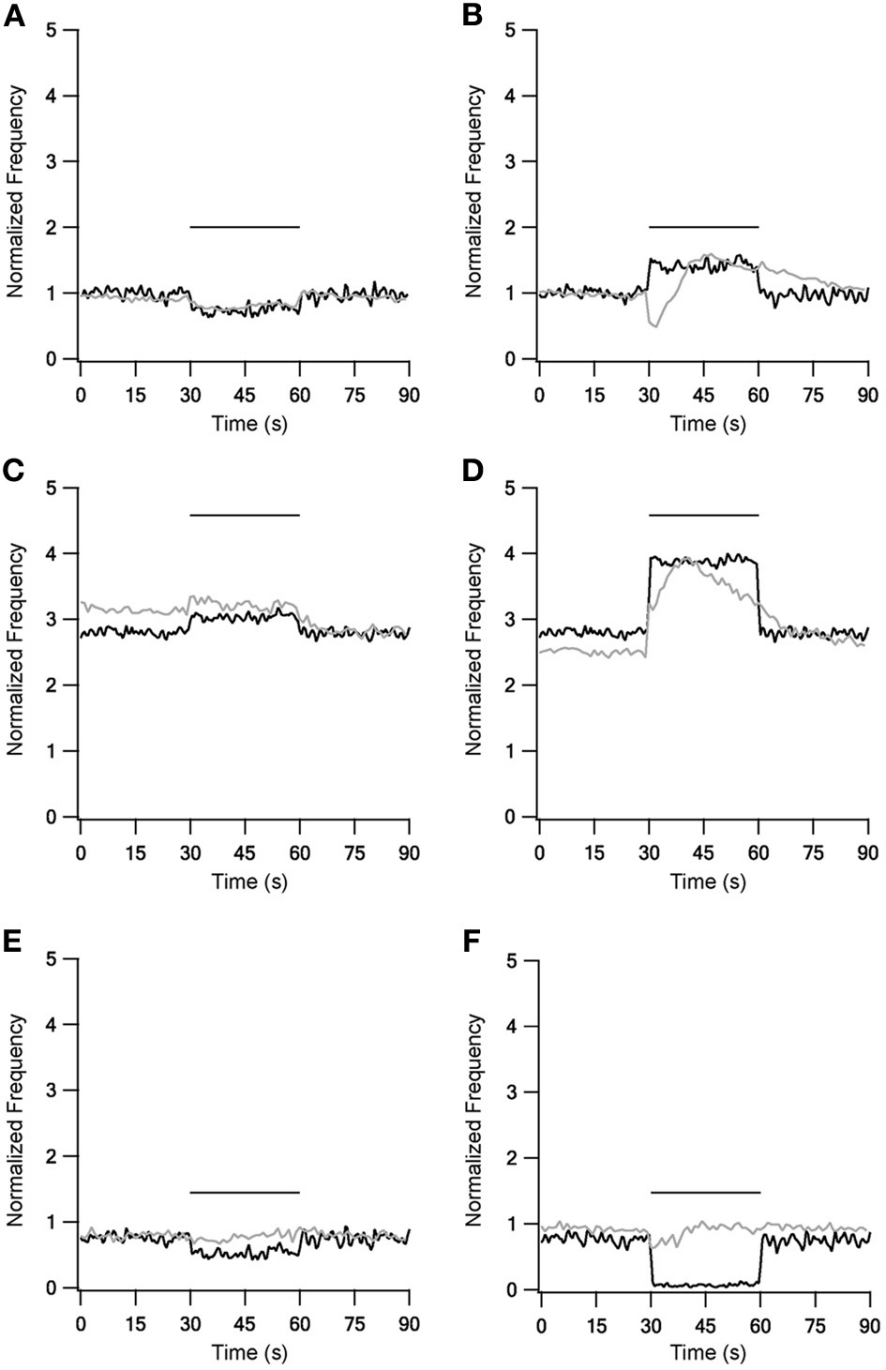
**Model and experiment results during low (LFS) and high frequency stimulation (HFS).** Response frequency over time normalized to mean prestimulus frequency under control conditions. Left column shows LFS results under control conditions **(A)**, during blockade of inhibition **(C)** and blockade of excitation **(E)**. Right column shows HFS results under control conditions **(B)**, during blockade of inhibition **(D)** and blockade of excitation **(F)**. Black traces, model results; gray traces, normalized population average recorded from GP neurons *in vitro* (Bugaysen et al., 2011). Horizontal bars indicate the stimulation period. In these experiments inhibition was blocked with 50 μM bicuculline and excitation with 15 μM CNQX and 50 μM APV.

Experimental firing rate changes during repetitive 10 Hz stimulation could be reproduced using these fast time constants (Figure 2, left column). Stimulation under control conditions slightly decreased the firing frequency (Figure 2A). A blockade of inhibitory synapses equivalent to *in vitro* application of bicuculline caused a marked increase of the spontaneous firing rate and a further increase during stimulation (Figure 2C). Blockade of excitatory synapses equivalent to *in vitro* application of APV and CNQX slightly decreased the spontaneous firing rate, while stimulation during the blockade caused an additional decrease. These phenomena were not observed experimentally (Figure 2E). Unlike the results obtained with LFS, the model had difficulty in capturing the effects of repetitive 40 Hz stimulation (Figure 2 right column). In all three conditions HFS induced a constant change in the firing frequency. This also contrasted with the *in vitro* results that showed complex modulation of the firing rate.

Since the model using fast forms of STP was unable to reproduce all experimental results, slower STP dynamics were introduced into the various synapses. These slow kinetics were set to be roughly on the expected order of magnitude that may correspond to the observed experimental time course. Thus, they may reflect not only STP but other cellular processes such as channel adaptation or intracellular calcium buildup. Slow depression with a time constant of 20 s was added to GP-GP synapses, while STN-GP dynamics were augmented and given a slower depression with time constants of 8 and 250 s respectively. A previous study showed that Str-GP synapses were completely depressed during HFS (Rav-Acha et al., 2005), thus Str-GP synapses were not included in 40 Hz stimulations and were not implemented with slower forms of STP.

**Figure 3:**
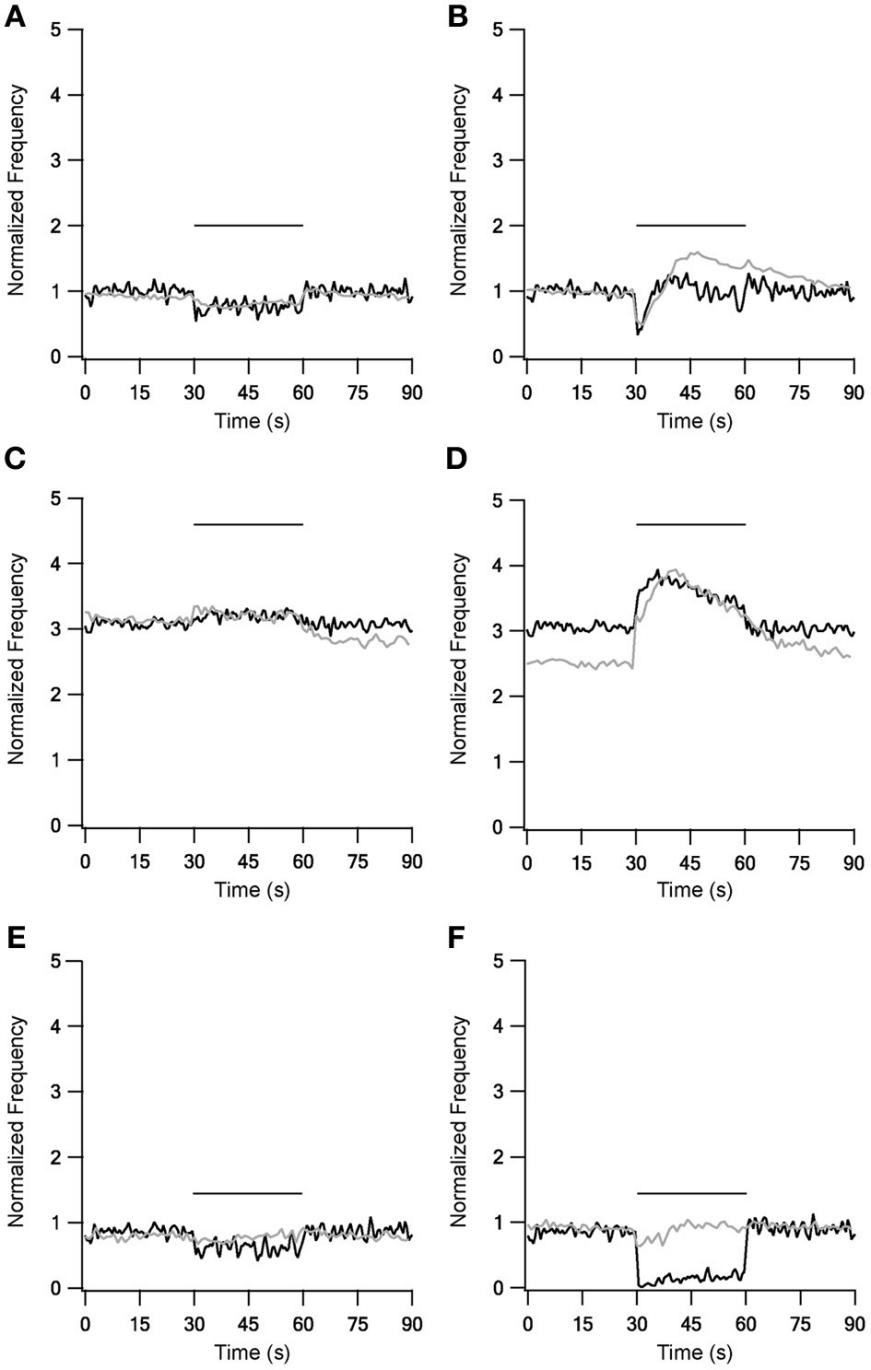
**Model and experimental results during LFS and HFS after adding slow kinetics to GP-GP and STN-GP synapses.** Left column shows LFS results under control conditions **(A)**, under blockade of inhibition **(C)** and blockade of excitation **(E)**. Right column shows HFS results under control conditions **(B)**, under blockade of inhibition **(D)** and blockade of excitation **(F)**. Plotted as in Figure 1. Black traces, model results; gray traces, normalized population average recorded from GP neurons *in vitro* (Bugaysen et al., 2011). Horizontal bars indicate the stimulation period. In these experiments inhibition was blocked with 50 μM bicuculline and excitation with 15 μM CNQX and 50 μM APV.

LFS results were not influenced by the new STP dynamics (Figure 3, left column), but during HFS most of the results were greatly improved (Figure 3, right column). With slower forms of STP the model succeeded in generating complex rate changes resembling the *in vitro* results during 40 Hz stimulation under control conditions (Figure 3B). The model faithfully captured the initial decrease in firing frequency but the following increase was smaller than observed experimentally. Results during inhibitory blockade highly resembled the *in vitro* results during stimulation but the model had less success reconstructing the after-stimulation effects (Figure 3D). The model failed to improve the results during blockade of excitation (Figure 3F). Overall, as the slow STP dynamics in STN-GP and GP-GP synapses significantly improved the model results, the model predicts the existence of slower plasticity dynamics in the GP that have not as yet been observed.

Following the addition of slow synaptic kinetics, the main differences between the model and the experimental results were: (1) the after-stimulation effects (Figures 3B,D) and (2) a lower firing rate during stimulation (Figure 3). These differences led us to add a mechanism implementing type 1 metabotropic glutamate receptors (mGluRs1). These are part of the metabotropic glutamate receptors located on GP neurons (Testa et al., 1994, 1998; Hanson and Smith, 1999; Smith et al., 2001; Marino et al., 2002; Poisik et al., 2003; Kaneda et al., 2007). mGluRs1 activation depolarize the membrane potential of GP neurons during and after stimulation (Poisik et al., 2003; Kaneda et al., 2007). Since STN-GP synapses were the only synapses in the model activated by glutamate, the mGluRs were merely added to their mechanisms.

**Figure 4:**
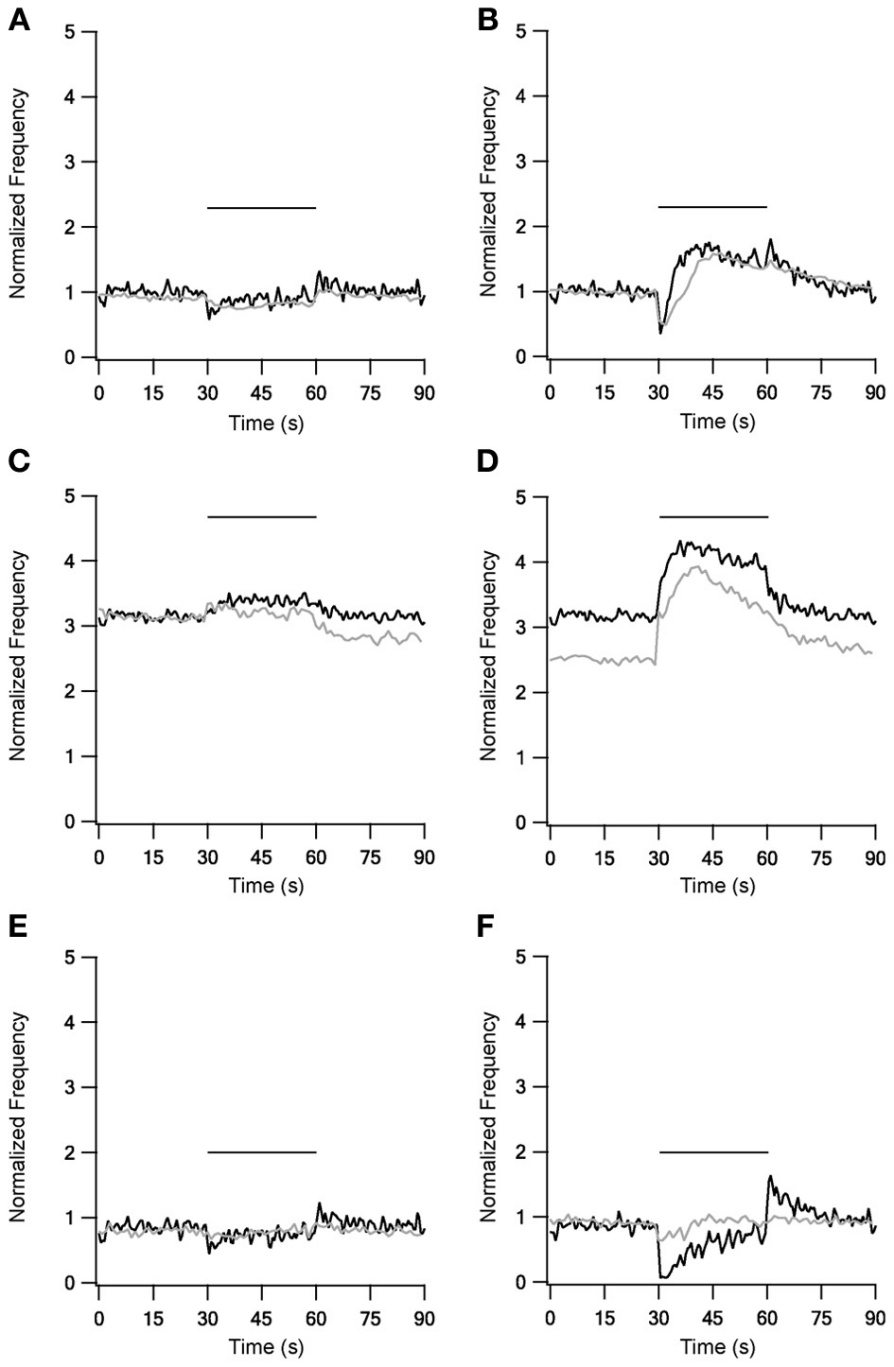
**Model and experimental results during LFS and HFS after adding mGluRs1 to STN-GP synapses.** Left column, results of LFS under control conditions **(A)**, during blockade of inhibition **(C)** and blockade of excitation **(E)**. Right column, HFS results under control conditions **(B)**, during blockade of inhibition **(D)** and blockade of excitation **(F)**. Black traces, model results; gray traces, normalized population average recorded from GP neurons *in vitro* (Bugaysen et al., 2011). In these experiments inhibition was blocked with 50 μM bicuculline and excitation with 15 μM CNQX and 50 μM APV. Plotted as in Figure 1 Horizontal bars indicate the stimulation period.

The addition of mGluRs kinetics improved both LFS and HFS results (Figure 4), with the improvement during HFS being more significant. 10 Hz stimulation under control conditions (Figure 4A) caused the firing rate to first decrease, but shortly afterwards it returned almost to the prestimulus level. Additionally, unlike the first two versions of the model, in this model firing rate increased slightly at the end of stimulation, an increase also observed experimentally. This version of the model with mGluRs also improved the results with blockade of inhibition and excitation, mainly by elevating the firing frequency during stimulation (Figures 4C,E). With 40 Hz stimulation, the results were markedly improved under control conditions (Figure 4B); after an initial decrease firing rate increased significantly for tens of milliseconds, remaining elevated after the end of stimulation. Under blockade of inhibition (Figure 4D) the mGluRs addition caused the firing rate to decay gradually after the end of the stimulation. Finally, firing rate increased during stimulation under blockade of excitation (Figure 4F), returning to almost the prestimulus firing rate. Even though these effects were not identical to the experimental results they showed a similar tendency. However, the induction of mGluRs also resulted in after-stimulation effects not observed experimentally.

**Figure 5:**
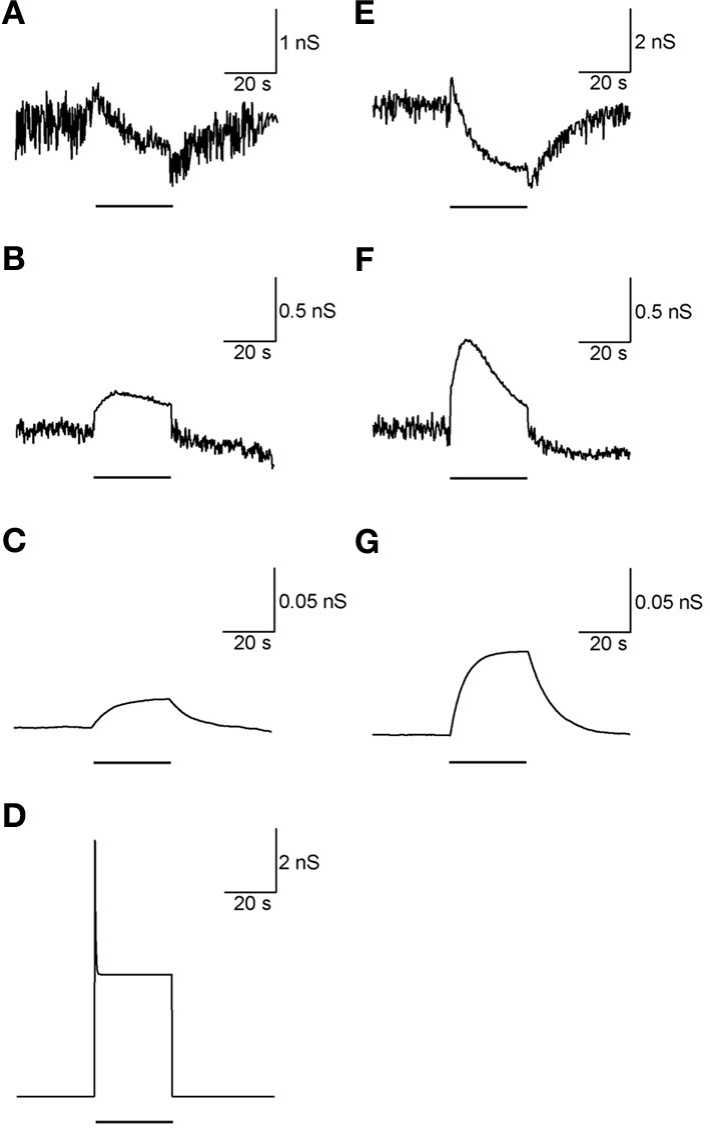
**Synaptic conductance during LFS and HFS. (A–D)** Synaptic conductance during 10 Hz stimulation of GP-GP synapses **(A)**, STN-GP ionotropic synapses **(B)**, STN-GP metabotropic synapses **(C)** and Str-GP synapses **(D)**. **(E–G)** Synaptic conductance during 40 Hz stimulation of GP-GP synapses **(E)**, STN-GP ionotropic synapses **(F)** and STN-GP metabotropic synapses **(G)**. Horizontal bars indicate the stimulation period.

**Figure 6:**
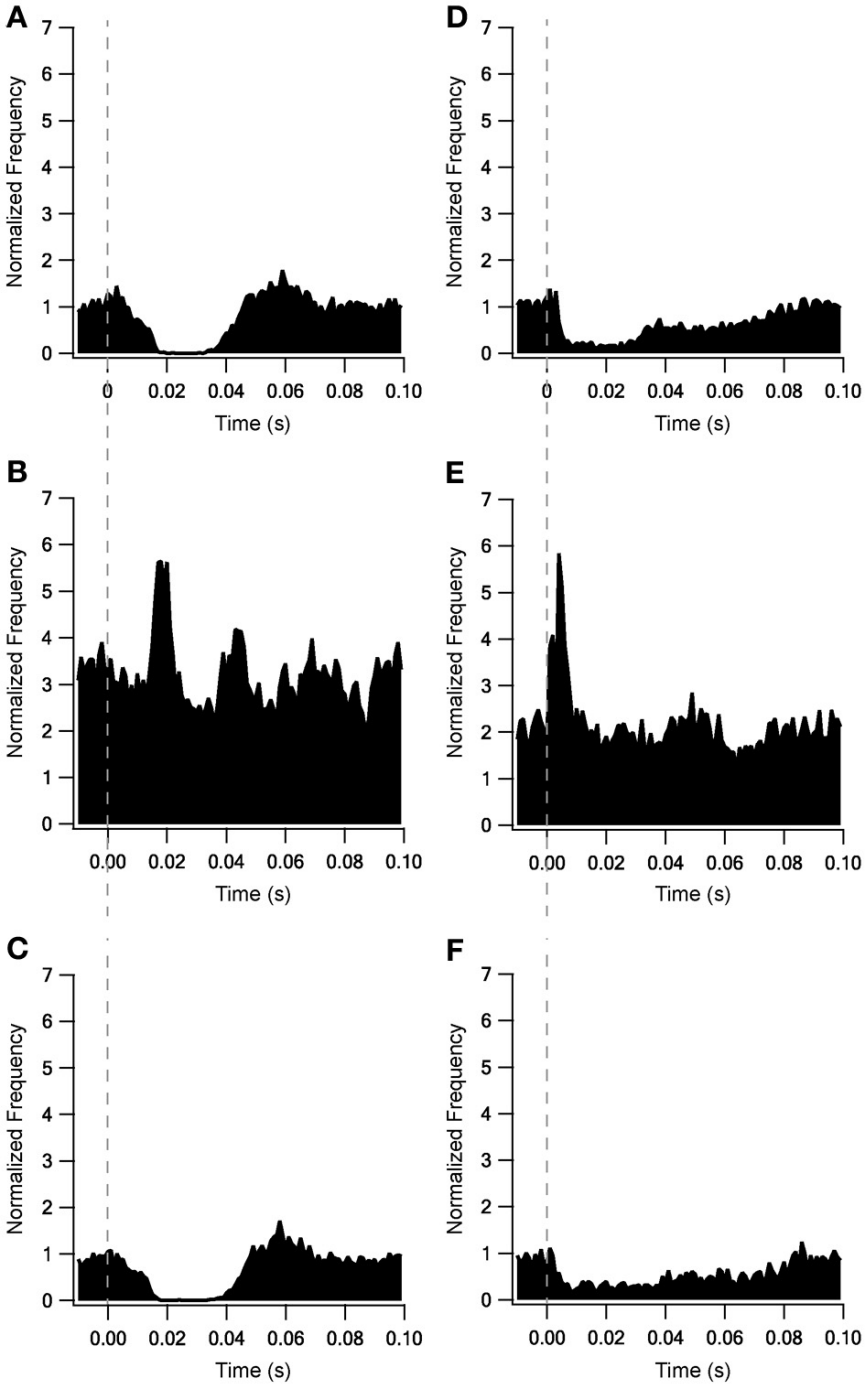
**Locking on to the stimulus during 10 Hz stimulation of model and experimental results.** Model results under control conditions **(A)**, blockade of inhibition **(B)** and blockade of excitation **(C)**. *In vitro* results from Bugaysen et al. (2011). **(D)**, Population PSTH of GP neurons in normal extracellular solution (*n* = 38). **(E)**, Population PSTH of GP neurons after applying 50 μM bicuculline to the extracellular solution (*n* = 17). **(F)**, Population PSTH of GP neurons after applying 15 μM CNQX and 50 μM APV to the extracellular solution (*n* = 14). The dashed vertical line marks the beginning of the stimulation pulse.

Adding the metabotropic receptors was the final step in the construction of our model. Figure 5 illustrates the conductivity of all synapses during LFS (Figures 5A–D) and all synapses except Str-GP synapses during HFS (Figures 5E–G), since Str-GP synapses were excluded during 40 Hz stimulation. After reaching the final model configuration we examined its ability to reconstruct rapid changes in firing pattern, including locking to the stimulus. Even though the model was fine-tuned based on the results of slow firing rates, similar rapid firing pattern effects were reproduced and the results under all three conditions included features resembling the experimental results (cf. Figures 6A–F). These results consisted of a decrease in firing rate immediately after the stimulus, which later returned to baseline. The recovery of the firing rate in the model was faster than observed experimentally. With blockade of inhibition (Figure 6B) the firing rate increase immediately after the stimulus, later decreasing and fluctuating around the prestimulus firing rate, similar to the experimental results (Figure 6E).

The addition of slow time constants to STN-GP and GP-GP synapses, as well as the addition of metabotropic receptors to STN-GP synapses, had a marked influence on the model results. Thus, the sensitivities of four parameters characterizing the various features of the slow dynamics were tested (see methods). We examined three parameters which characterize the STN-GP synapses - augmentation time constant (τ_*A*_), slow depression time constant (τ_*D*_Slow__) and the reaction rate constant describing the metabotropic receptor decay (*Kb*_*R*_)—and the depression time constant (τ_*D*_) characterizing GP-GP synapses. Random values of these parameters were sampled from different normal distributions when the distribution mean for each parameter reached its final value in the model and the SD was 20% of the mean for τ_*D*_Slow__, *Kb*_*R*_, τ_*D*_, and 30% for τ_*A*_.

**Figure 7:**
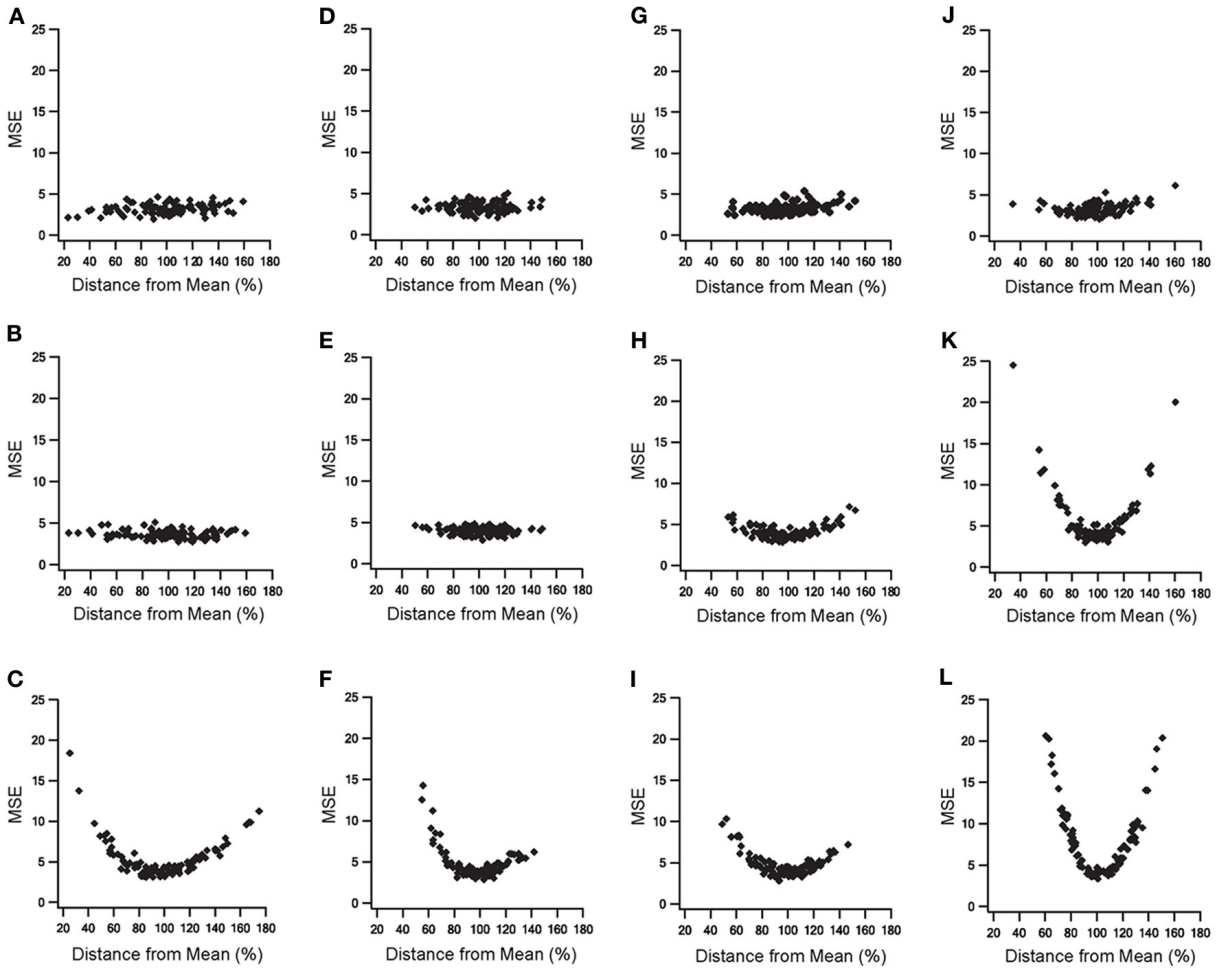
**Parameter sensitivity analysis of the parameters characterizing slow dynamics of STN-GP and GP-GP synapses.** Means square errors (MSE) vs. percent distance from the means. Effects of changing time constant of STN-GP augmentation **(A–C)**, effects of changing time constants of slow depression of STN-GP **(D–F)**, effects of changes in rate constant of decay reaction in metabotropic receptors **(G–I)** and effects of changing time constant of GP-GP depression **(J–L)**. Top row, errors during 10 Hz stimulation; middle row, effects on firing rate during 10 Hz stimulation; bottom row, effects on the firing rate during 40 Hz stimulation.

Figure 7 illustrates the results of the parameter sensitivity analyses for these four parameters. During 10 Hz stimulation the population PSTH results of all parameters exhibited similar errors for the various values sampled (Figure 7, top row). This indicated that these parameters mildly influenced firing pattern during LFS. These findings are reasonable since the population PSTH time windows were 100 ms while the slow dynamics time constants ranged from several to hundreds of seconds. Firing rate results during 10 Hz stimulation differed for the four parameters (Figure 7, middle row). Changing the values of the augmentation and the slow depression of STN-GP synapses resulted in similar errors for the different sampled values (Figures 7B,E), indicating that these parameters had no impact during LFS. That is, these results fit the previous results showing no change in the firing rate during LFS after adding slow time constants to STN-GP synapses (Figure 3, left column).

However, changing the reaction rate constant and the GP-GP depression time constant resulted in a greater error, as the sampled values were farther from the mean (Figures 7H,K) indicating that the model was sensitive to changes in these parameters. The results for the reaction rate constant of the metabotropic receptors correlated with the addition of these receptors having some influence on the model results during LFS (Figure 4, left column).

The results for the depression time constant of the GP-GP synapses showed a marked influence on the model results, even though this kind of influence was not apparent during LFS (Figure 3, left column). During 40 Hz stimulation changes in all four parameter values produced greater error, showing a clear influence on the model results (Figure 7, bottom row). This fitted the finding that both slower synaptic dynamics and metabotropic receptors greatly improved the model results during HFS (Figures 3, 4 right columns).

DBS treatment for Parkinson's disease is only therapeutic at frequencies above 100 Hz (Dostrovsky and Lozano, 2002), while LFS has no effect on the symptoms or even worsens them (Rizzone et al., 2001; Moro et al., 2002). An *in vitro* study showed that the activity of STN neurons is differently modulated by HFS and LFS (Garcia et al., 2003). Thus we tested the response of GP neurons to stimulation at various frequencies to determine whether HFS and LFS also have different effects on GP activity.

**Figure 8:**
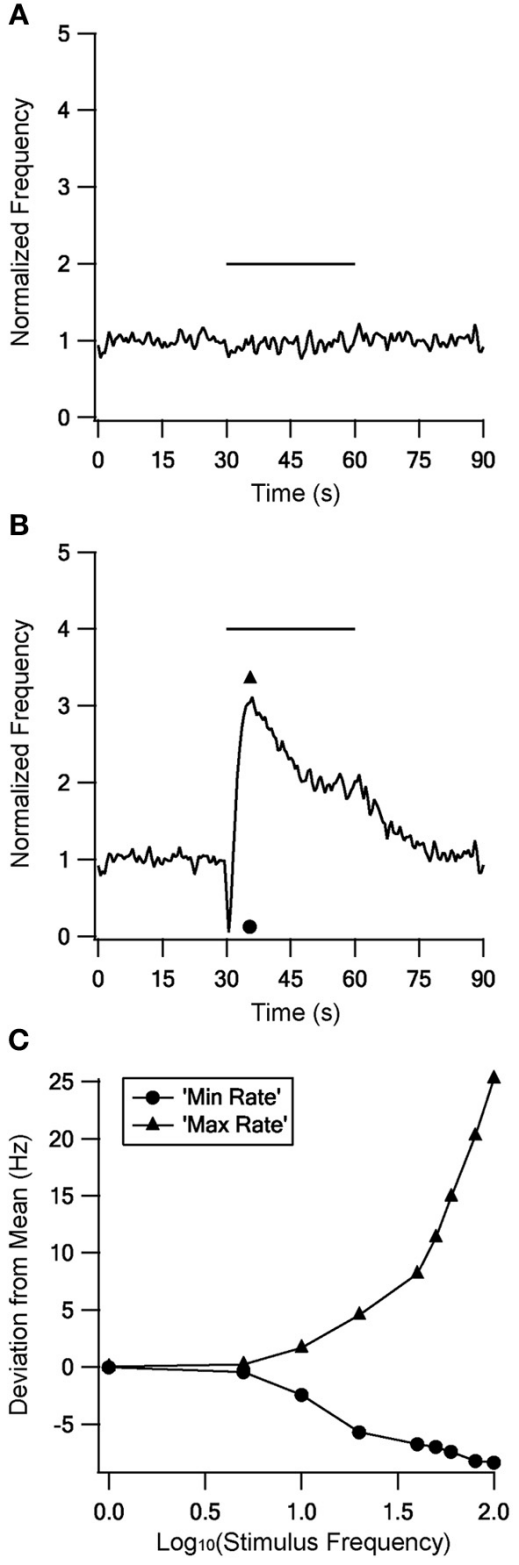
**The model response to stimulation at different frequencies.** The model response to stimulation at 5 Hz **(A)** and 100 Hz **(B)** to illustrate the responses to LFS and HFS respectively. **(C)** Minimal (circles) and maximal (triangles) firing rates observed during each stimulus frequency.

No changes were found during stimulation at frequencies up to 10 Hz; Figure 8A gives an example during 5 Hz stimulation. In contrast, there were marked changes in firing rate during stimulation at or above 20 Hz. Above 40 Hz the response became biphasic, with an initial decrease in firing rate followed by an increased firing rate that decayed during the stimulation but returned to the mean prestimulus firing rate only tens of seconds after the end of stimulation. Figure 8B shows a response to 100 Hz stimulation. To quantify the firing rate differences during LFS and HFS we compared the minimal and maximal firing rates observed with each stimulus frequency (Figure 8C). This quantification emphasized that LFS had little influence on the firing rate; up to 10 Hz stimulation elicited no or only small differences between the minimal and the maximal firing rate. In contrast, HFS resulted in large differences which increased with stimulus frequency.

## Discussion

This main objective of this study was to qualitatively simulate GP activity during low (LFS) and HFS, while emphasizing the importance of STP dynamics at the three main synapses of the GP; synapse with neurons from the striatum (Str-GP), from the subthalamic nucleus (STN-GP) and synapses between GP neurons (GP-GP). We constructed a model imitating the activity of a simple postsynaptic neuron connected to a small input network.

The model presented in this work aimed at qualitatively investigating the role of STP on firing of GP neurons. Thus, the main focus of the model was the description of synaptic dynamics while the firing dynamics of the postsynaptic neuron were simulated using a simple Hodgkin-Huxley like model (Pospischil et al., 2008). This simplified postsynaptic model does not take into account the large number of ion channels expressed in GP neurons nor their contribution to slow postsynaptic integration. It is quite likely that the slow time constants we added to the synapse in order to better simulate the response of the neuron to repetitive stimulation may not be a synaptic property but one stemming from postsynaptic channel activity. Indeed both experiments and modeling have shown substantial contribution of calcium activated channels to the firing of GP neurons (Deister et al., 2009). Thus, the current simulations present a set of thought experiments aimed at identification of the possible role of STP in GP firing rather than a full model of the postsynaptic neuron. The first model implemented with the fast STP dynamics reported for Str-GP (Rav-Acha et al., 2005) and STN-GP (Hanson and Jaeger, 2002) synapses failed to reconstruct *in vitro* HFS effects (Figure 2) (Bugaysen et al., 2011). This result focused the need to address longer time scales when considering the effect of prolonged stimulation of GP neurons. Thus, slower STP dynamics were introduced; depression was added to GP-GP synapse dynamics and both depression and augmentation added to the STN-GP synapse dynamics. The addition of these synaptic processes greatly improved the model results during HFS (Figure 3), suggesting the existence of slower STP dynamics at GP synapses that have not yet been isolated in biological preparations.

Since slow STP dynamics have a great influence during prolonged HFS it is especially important to consider them in the GP, since the GP serves as a target for high frequency DBS in the attempt to treat symptoms of Parkinson's disease (Yelnik et al., 2000; Dostrovsky et al., 2002; Bar-Gad et al., 2004; Vitek et al., 2004). Under normal conditions GP neurons do not appear to exhibit correlated activity (Nini et al., 1995; Bar-Gad et al., 2003; Goldberg and Bergman, 2011), but correlation was observed in 20% of paired GP neurons in monkey and rat Parkinson's models (Nini et al., 1995; Mallet et al., 2008; Bronfeld et al., 2010; Goldberg and Bergman, 2011). Synaptic depression can decorrelate neuron activity (Abbott and Regehr, 2004), thus the depression predicted by our model at GP-GP synapses could cause decorrelation during HF-DBS treatments of the GP and STN nuclei. Since both GP and STN DBS (Benabid, 2003) and GP DBS (Vitek et al., 2004) alleviate Parkinson's symptoms, it is tempting to speculate that decorrelation derived from synaptic depression is one of the mechanisms underlying the therapeutic effects of DBS.

Implementation of metabotropic glutamate type 1 receptors, which have been found in GP neurons (Testa et al., 1994, 1998; Hanson and Smith, 1999; Marino et al., 2002), further improved the model results. Their major contribution was to the reconstruction of after-stimulation effects (Figure 4). Thus, our model predicts that these after-stimulation effects are due to the activation of mGluRs and not of ionotropic synapses, agreeing with previous studies (Poisik et al., 2003; Kaneda et al., 2007).

All model responses to HFS derived from activation of only STN-GP and GP-GP synapses, without any activation of Str-GP synapses. This implies Str-GP synapses have no impact on GP activity during HFS and, indeed, Rav-Acha et al. (2005) showed that Str-GP synapses were completely depressed above 33 Hz stimulation. In the final configuration of the model STN-GP synapses accounted for 30% of the inputs, Str-GP synapses for 15% and GP-GP synapses for 55%. Histochemical analyses show that STN-GP synapses account for less than 20% of the inputs, 80% of inputs arise from Str-GP synapses and the remainder from GP-GP synapses (Kita and Kitai, 1994; Shink and Smith, 1995; Kita, 2007; Sadek et al., 2007). These discrepancies could result from the properties of the *in vitro* slices from which the physiological data set for reconstruction was obtained. As these brain slices included mainly the GP nucleus, it is plausible that the GP-GP connections in the slice were much better preserved than the other connections. This could have biased the original data set away from the conditions in which the histochemical results were obtained.

A higher baseline firing rate of STN neurons than the 6 Hz frequency used here has been reported in a number of studies (Nakanishi et al., 1987; Beurrier et al., 1999; Do and Bean, 2003; Hallworth and Bevan, 2005). Hanson et al. (2004) found sodium channels near STN boutons along GP dendrites, and these channels amplified the depolarization generated by activation of STN-GP synapses. Elevating the baseline firing rate of STN neurons and/or increasing the EPSP size in STN-GP synapses to fit these studies would probably reduce the number of STN neurons, bringing their number closer to the anatomical results.

Str-GP and GP-GP synapses were both implemented with IPSPs of 0.33 mV. Str-GP synapses are located mainly on GP dendrites, while GP-GP synapses are located only on the soma. Moreover GP-GP synapses are larger than Str-GP synapses (Falls et al., 1983; Kita, 1994, 2007; Shink and Smith, 1995). Therefore, the IPSPs generated after activation of Str-GP synapses could be smaller than those generated by GP activation of GP-GP synapses. Smaller IPSPs would most likely increase the number of Str neurons, closer to the anatomical results.

Stimulating the model with frequencies from 1 to 100 Hz resulted in different responses to LFS (up to 10 Hz) and HFS (20 Hz and above) (Figure 8). Different responses to LFS and HFS have also been observed in the STN (Garcia et al., 2003). DBS treating for Parkinson's disease is only therapeutic at high frequencies and has no impact at low frequencies (Rizzone et al., 2001; Dostrovsky et al., 2002; Moro et al., 2002). DBS efficiency with HFS may thus result from the strong influence of HFS on the firing rate and firing pattern of both the GP and the STN, changes which are not apparent during LFS. The results of the model predict the existence of new STP dynamics and that GP neurons respond differentially to low and HFS. Testing these predictions in biological preparations will further our understanding on GP activity in the normal and pathological state, as well as during DBS treatment in Parkinson's disease.

### Conflict of interest statement

The authors declare that the research was conducted in the absence of any commercial or financial relationships that could be construed as a potential conflict of interest.
